# Digital health interventions for cervical cancer care: A systematic review and future research opportunities

**DOI:** 10.1371/journal.pone.0296015

**Published:** 2023-12-15

**Authors:** Md Abdur Razzak, Muhammad Nazrul Islam, Md Shadman Aadeeb, Tasfia Tasnim

**Affiliations:** 1 Faculty of Science and Technology, Bangladesh University of Professionals, Dhaka, Bangladesh; 2 Department of Computer Science and Engineering, Military Institute of Science and Technology, Dhaka, Bangladesh; University of Jos Faculty of Medical Sciences, NIGERIA

## Abstract

**Background:**

Cervical cancer is a malignancy among women worldwide, which is responsible for innumerable deaths every year. The primary objective of this review study is to offer a comprehensive and synthesized overview of the existing literature concerning digital interventions in cervical cancer care. As such, we aim to uncover prevalent research gaps and highlight prospective avenues for future investigations.

**Methods:**

This study adopted a Systematic Literature Review (SLR) methodology where a total of 26 articles were reviewed from an initial set of 1110 articles following an inclusion-exclusion criterion.

**Results:**

The review highlights a deficiency in existing studies that address awareness dissemination, screening facilitation, and treatment provision for cervical cancer. The review also reveals future research opportunities like explore innovative approaches using emerging technologies to enhance awareness campaigns and treatment accessibility, consider diverse study contexts, develop sophisticated machine learning models for screening, incorporate additional features in machine learning research, investigate the impact of treatments across different stages of cervical cancer, and create more user-friendly applications for cervical cancer care.

**Conclusions:**

The findings of this study can contribute to mitigating the adverse effects of cervical cancer and improving patient outcomes. It also highlights the untapped potential of Artificial Intelligence and Machine Learning, which could significantly impact our society.

## Introduction

Cervical cancer is the fourth most frequent malignancy among women, while accounting for 341,831 deaths in 2020 [1]. In underdeveloped nations, it is the most frequent kind of cancer. The most sensitive location for forming tumors that can later lead to cancer is the squamocolumnar junction located in the cervix [2]. Invasive cervical cancer accounts for around 75% of all occurrences of prevalent malignancies while cervical cancer is caused by the Human Papillomavirus (HPV) 95 percent of the time being the most common cause of cervical cancer [3]. HPV infection is normally eliminated from the body on its own, but it can occasionally develop into a premalignant condition that can lead to cancer over time [4]. However, it might happen in less than a year for 10% of women [5]. Again, a significant percent of the 270,000 fatalities due to cervical cancer occurred in low and middle-income nations, demonstrating that low and middle-income countries are at higher risk than developed countries due to a lack of systematic screening and HPV vaccination programs [6]. HPV infection, smoking, and a history of sexually transmitted diseases are the most prominent causes of cervical carcinomas [7]. Low socioeconomic status, multiple sexual partners, and immunosuppression are some of the other minor factors [8].

Cervical cancer is curable, but if not caught early enough, it can lead to death in women. Reducing cervical cancer risk factors can help people who are at high risk of developing the disease [9]. Illiteracy and semi illiteracy, having low socioeconomic status, early marriage, and having a high parity are all common causes of HPV infection which leads to being affected by cervical cancer [10]. After radical surgery for cervical cancer, many women experience urological complications [11]. On top of that, some of them experience sexual dysfunctionalities which leads to emotional suffering [12]. Cervical cancer can be avoided and possibly cured if precautions are taken ahead of time. Some screening procedures are well-known for determining the presence of cervical cancer, such as cytology via Papinicolaou (Pap) smear, visual inspection by acetic acid (VIA), human papillomavirus–deoxyribonucleic acid (HPV–DNA), careHPV–DNA, and others [9].

Recent studies have shown that three key factors play a pivotal role in mitigating cervical cancer and its impacts which include the development of awareness, effective screening and proper treatment [13–15]. Research has shown that educational interventions augmented with fact-based awareness messages from established organizations significantly aid in spreading awareness and dissemination of information. Screening services equipped with state of the clinical approaches have been determinedto be capable of eliminating all instances of cervical cancer in a country [14]. Furthermore, recent surveys have shown that ensuring proper treatment facilities immediately after screening, scaling up treatment modalities and inclusion of digital interventions greatly increase the chances of survival of cervical cancer patients [16–18].

Digital intervention in cerviceal cacner refers to the use of advanced digital technologies to enhance health outcomes, patient care, and the efficiency of healthcare services for cervical cancer. In this context, our study emphasises the integration of mobile applications, machine learning (ML), and artificial intelligence (AI) as key components of digital intervention. Mobile applications are instrumental in this framework, offering accessible platforms for health monitoring, education, and engagement between patients and healthcare providers. They provide personalised healthcare services and enable effective management of health data in real time [16]. Further, ML and AI technologies are part and parcel in the analysis and interpretation of complex healthcare data. They facilitate predictive analytics, improve diagnostic accuracy, and support the creation of customised treatment plans. These technologies transform patient’s health data into meaningful insights, and thus significantly contribute to the advancement of digital healthcare interventions [17]. This combination of mobile apps, ML, and AI represents a holistic approach to digital intervention, highlighting the potential of technology in revolutionizing healthcare delivery and patient care.

Therefore, the objective of this review study is to systematically summarize and evaluate the current digital interventions for creating awareness, providing screening service as well as treatment facilities for cervical cancer patients worldwide. To attain this objective, 26 related articles and 9 numbers of mobile applications were meticulously reviewed following the systematic literature review approach. As suggested in [19], the contribution of this study can be highlighted as:

- An integrated synthesized view of the current state of the knowledge on digital interventions for cervical cancer patients.
- Explore the methodologies of the existing studies and their unique insights.
- Describe research insights, existing gaps, and future research directions.

The remaining section of this article is organized as follows: the related review studies and the review methodology are highlighted as presented in Materials and Methods section. The analysis and findings of the review data are discussed in Results section. Discussion section represents the main findings with the future research directions. This sections also highlights the limitations and provides concluding remarks.

## Materials and methods

### Review studies

In this section, related review studies are briefly discussed and analysed. Based on the analysis the contributions, shortcomings and research gap in the related studies were highlighted.

Xue et al. [20] conducted a systematic review and meta-analysis on studies where cervical cancer and breast cancer detection was done using deep learning (DL) based systems. The meta-analysis was conducted based on four criteria which included validation types, cancer types, imaging modalities and comparative performance of DL algorithms against professional clinicians. Furthermore, the quality of the selected studies was assessed using state of the art quality assessment techniques. This review paper identified several positive implications of using deep learning techniques in cervical cancer screening. The notable benefits of using Deep Learning based approaches mentioned by this paper included reduction of over-reliance on experienced clinicians and achievement of high diagnostic and screening performance. However, this research also identified several drawbacks among the reviewed articles which included methodological deficiencies, lack of sufficient clinical trials, poor study design and reporting, biased arguments and over estimation of algorithmic performances. Though this paper thoroughly focuses on deep learning based cervical cancer screening approaches and is successful in achieving its objectives, there are some limitations in this study. Firstly, this review article exclusively focuses on only DL based screening techniques. No review of screening techniques based on other approaches were done in this paper. Moreover, this research does not take into consideration the awareness and treatment aspects which are equally important for mitigating cervical cancer and its impact.

Peirson et al. [21] in their research conducted a review of several observational studies related to cervical cancer screening. This review article focused on three questions related to the effect of cervical cancer screening, varying screening interval and varying screening age. The review results showed that performing screening for cervical cancer for once in the entire lifetime significantly reduces the risks associated with cervical cancer. Furthermore, the review results suggested that there is no optimal age for performing cervical cancer screening. Moreover, a screening interval of 5 years ensures substantial protective effects against cervical cancer. However, despite exploring several factors associated with screening, this research did not take into consideration the treatment and awareness measures associated with cervical cancer. Furthermore, this work exclusively focuses on screening studies, excluding consideration for digital interventions in cervical cancer such as mobile applications and the application of artificial intelligence (AI) and machine learning (ML).

Lee et al. [22] in their review work, investigated several educational tools including the use of mobile apps in increasing preventive behaviours and cancer literacy among women. The influences of these rolls were assessed based on four domains which were health behaviour, attitude towards behaviour change, self-efficacy of behaviour, and cervical cancer literacy. The results of this review article indicated that the augmentation of traditional learning and awareness approaches with mobile app assisted approaches can serve as an innovative and effective approach for increasing cervical cancer awareness and knowledge among women.

Rossman et al. [23] in their research reviewed several studies related to cervical cancer control. The reviewed articles focused on screening, treatment and patient education using digital interventions. This research identified several research gaps related to cervical cancer which are required to be thoroughly addressed. Firstly, as per this research, there is insufficient evidence regarding the effectiveness of digital health strategies for cervical cancer screening and treatment. Secondly, future cervical cancer related research works should adopt more rigorous study designs for increasing effectiveness. Thirdly, the reporting of digital health strategies in the literature is required to be improved and finally expansion of research is required in the lower and middle income countries in Asia and South America to attain more relevant data from a wider variety of sources.

Shanthi et al. [24] conducted a comprehensive review of several algorithms for cervical cancer screening with the help of pap smear microscopic images. The authors of this article encouraged the adoption of machine learning based algorithms in cervical cancer diagnosis after reviewing several machine learning algorithms utilised in different studies. Furthermore, this research also highlighted the challenges faced during automated diagnosis of cervical cancer which included noise removal, segmentation for extracting the region of interest, dataset collection, etc. Similar research was conducted by William et al. where it was identified that machine learning tools can achieve high accuracy when diagnosing cervical cancer. Moreover, the authors also suggested the combination of several machine learning algorithms (for example K-nearest neighbours with support vector machines) in order to improve diagnosis. This research also revealed some of the pitfalls associated with cervical cancer screening using machine learning, for example, it has been found that in some classes of cells, the diagnosis of cervical cancer results in low accuracy.

Bhochhibhoya et al. [25] focused on reviewing research that focused on increasing cervical cancer knowledge and awareness. The study concluded that mHealth based interventions are useful for increasing overall knowledge of the general mass. Furthermore, mHealth based interventions also increase the number of screening tests for cervical cancer. But in order to successfully achieve the objectives, an mhealth application needs to increase the frequency of information dispatching to the users. Moreover, the information is dispatched to the users. Moreover, the information should be imparted using several mediums including texts, phone calls and mails. However, this research identified breaching of privacy as one of the core concerns associated with mHealth applications. In another study, Champin et al. [26] reviewed 16 studies to explore the usefulness of the smartphone for detecting uterine cervical cancer.

Sarah et al. [27] reviewed several cervical cancer screening approaches which were augmented by digital interventions as well. The reviewed systems included digital papilloma virus detection systems and digital colposcopy which are widely used for cervical cancer screening. This research also focused on reviewing the practices adopted for cervical cancer treatment as well which includes gas based cryotherapy, thermoablation, HPV vaccination, etc.

Based on the review of the related review articles, it has been found that several prior researches focused on reviewing cervical cancer awareness, screening and treatment approaches. These researchers and authors of these articles were successful in fulfilling their objectives and provided useful findings and implications which will aid future research.

However, very few of the reviews were conducted considering all the aspects associated with cervical cancer including raising awareness, screening and treatment. Very few reviews were done focusing on enhancing cervical cancer treatment using digital interventions. Furthermore, despite the development of several mobile apps for cervical cancer awareness, screening and treatment purposes, no research focused on analytically and empirically reviewing those. Furthermore, no research conceptualized a system for providing cervical cancer awareness, screening and treatment services, all in one platform despite the fact that the necessity of these services have been justified individually in different researches.

### Search strategy

Apart from the Google search engine, primary articles were searched in various sources to discover relevant literature for this review study such as Google Scholar, Springer Link, Scopus, IEEE Xplore, SpringerLink, PubMed, Dovepress, ACM Digital Library, etc. Several keywords and their synonyms were used to search the articles, for example, ‘Cervical cancer screening’, ‘human papillomavirus cervical cancer’, ‘Cervical cancer awareness illiterate women’, ‘Cervical cancer prevention’, ‘Cervical cancer diagnosis’, ‘Cervical cancer prediction using machine learning algorithms, ‘Cervical cancer detection using deep learning’, ‘Mobile application system for cervical cancer detection’, ‘Mobile app for Cervical cancer screening and awareness for women’, ‘Mobile app for the info of Cervical cancer in the underdeveloped country’, ‘Mobile application system using machine learning for screening, detecting and prevention of cervical cancer’, ‘digital system for cervical cancer screening and awareness in underdeveloped countries’, ‘education for cervical cancer awareness’, ‘cervical cancer pap test screening’. The search was carried out continuously during June to December 2022, while the articles published in the last 11 years (2011–2022) were included in the review. The selection of the time frame from 2011 to 2022 for this study was done mainly due to two primary considerations. Firstly, this period marks a significant era in healthcare, characterised by the rapid digitization of healthcare applications. The last decade has witnessed an unprecedented transformation in the healthcare sector, driven by technological advancements and digital innovation. This shift has fundamentally altered the landscape of healthcare delivery, making it a critical period for examination. Secondly, a decade-long study offers a robust temporal scope, allowing for a comprehensive analysis of trends, developments, and outcomes. This time frame not only provides a historical perspective but also ensures relevance to current and future applications in digital healthcare. As such, this duration is considered to observe the evolution of digital healthcare practices and their impacts, thereby enabling the transformation of our research into a state-of-the-art reference in the field. Different types of publications like conference articles, journals and various mobile applications were searched for finding the maximum number of related articles and systems already developed for this research.

### Inclusion and exclusion

The following criteria were used to select only relevant articles and apps for review: a) Article focuses to the design, development, adoption and uses of mobile applications or other digital systems for Cervical cancer screening, detection, awareness, and

- prevention by using machine learning or other methods
- Articles related to cervical cancer awareness for women through digital intervention.
- Studies published between 2011 to 2022.

Furthermore, the following exclusion criteria were used:

- Duplicate articles
- Articles that are not written in English
- Articles diverging from the primary focus of this review, that is, the design, development, adoption, and utilization of mobile applications or digital systems for cervical cancer screening, detection, and awareness
- Articles not focused on our research objective

### Study selection

**Fig 1:**
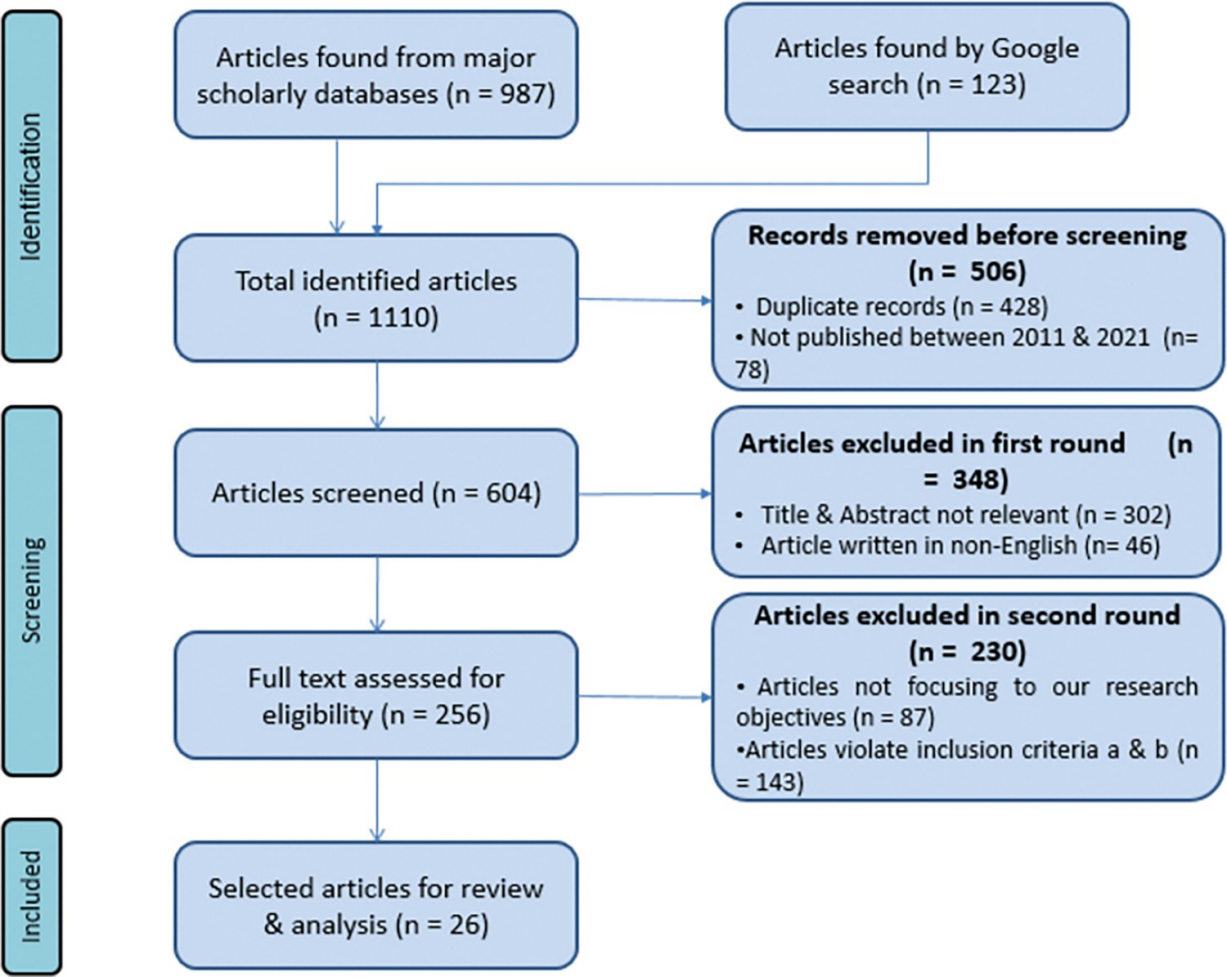
PRISMA flow chart for article selection.

A PRISMA flow diagram in Fig 1 shows the detailed study selection process following the inclusion and exclusion criteria. After searching from the stated scholar databases, a total of 1110 research studies were discovered including 123 studies found from Google. The article selection procedure began with the removal of 428 duplicated articles and the exclusion of another 78 articles that were not published between 2011 and 2022. During the second round of screening, articles were selected based on whether those were written in English and their title and abstract. 46 articles were excluded as those were written in non-English language and 302 articles were excluded as their titles and abstracts were not relevant. After this round of the selection process, a total of 256 articles were included. Finally, after reading the abstract and introduction 87 articles were excluded since those did not match with the research objective and 143 were excluded as those were irrelevant with the inclusion criteria. After applying the indicated inclusion-exclusion process in three steps, the study review included a total of 26 research articles.

### Selection of related mobile apps

This research involved analysing different healthcare applications from the Google Play Store and Apple App Store. We focused on apps with high download rates and user ratings. A total of 19 apps were initially selected for the review process. Out of these, after examining the relevance, 9 of mobile applications were meticulously reviewed and analysed. The evaluation process involved rigorous operational testing of these applications. This was executed by running each application under controlled conditions to examine their functionality, user interface, features, and overall performance. Special attention was given to how these applications cater to the needs and challenges specific to Cervical Cancer care and management. The results of the app analysis have been presented in the subsection “Mobile applications in Cervical Cancer”. The review process not only provided an understanding of the current state of mobile healthcare applications but also offered a glimpse into the evolving landscape of digital health solutions, their usability, and their impact on healthcare delivery.

### Data extraction and analysis

**Fig 2:**
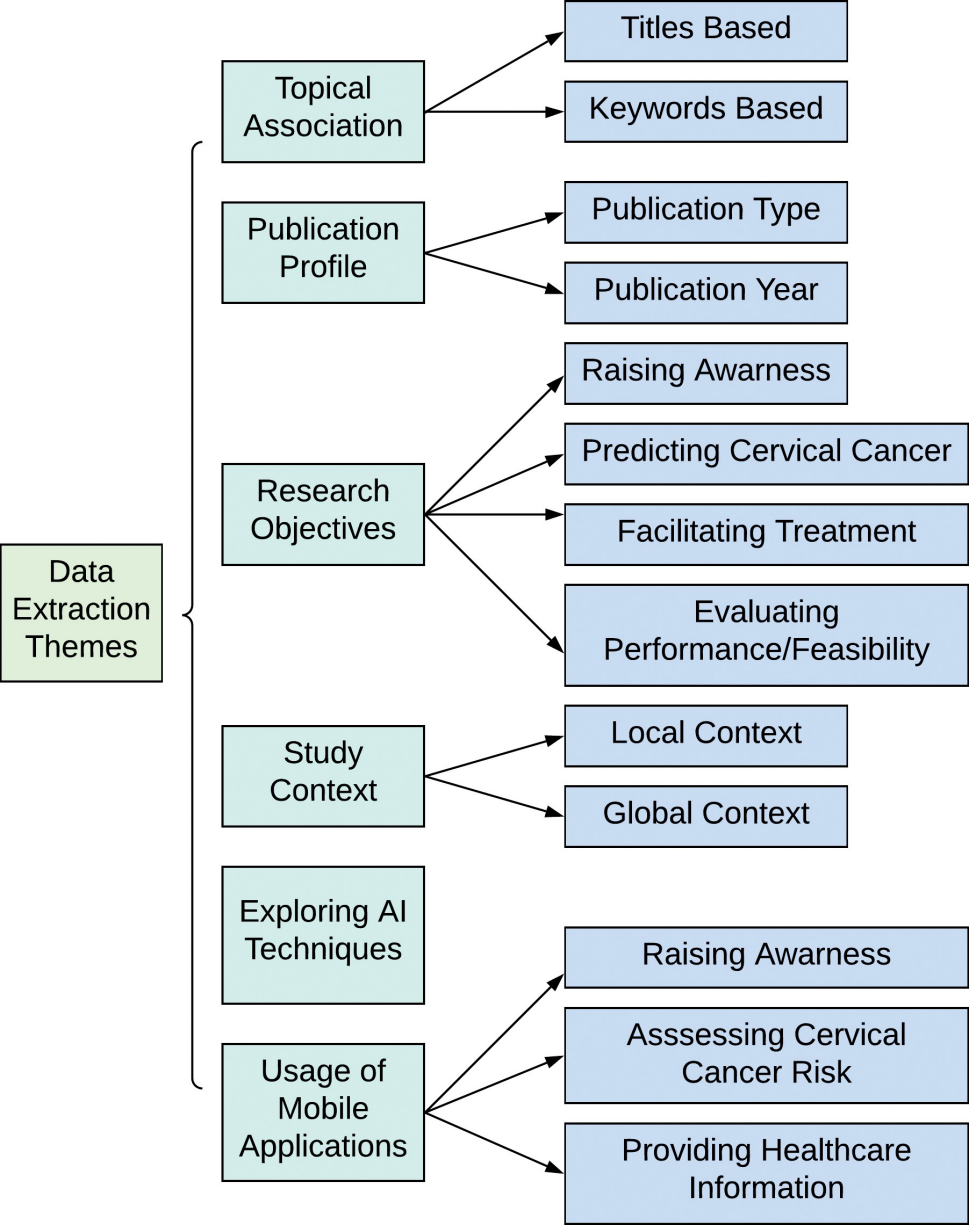
Themes for data extraction.

To extract the core characteristics and substantial information from the selected articles in a systematic manner, six prominent themes were considered as shown in Fig 2. The types of data obtained from the reviewed articles can be easily perceived with the help of these six themes. The themes are succinctly discussed as follows.

- *Topical Association*: In this theme, the association and the relevance among the reviewed articles were investigated by assessing the word clouds, which are generated by considering the articles’ titles and keywords.
- *Publication Profile*: This theme delineates the publication year of the reviewed articles and the types of the papers like journal papers, conference papers, etc.
- *Research objectives*: This theme classified the key objectives of the reviewed articles into four groups, emphasizing the key perspectives, covered in the domain of cervical cancer research. These groups include: raising awareness, predicting cervical cancer, facilitating treatment, and evaluating performance/feasibility.
- *Study context*: The selected research articles are from varied contexts. For example, some studies are from a specific country context, while other studies are carried out considering the global perspective. Thus, this theme classified the country context of the reviewed articles, into two groups: local context and global context.
- *Exploring the AI techniques*: The different AI techniques adopted by the different studies are portrayed under this theme, accentuating the frequency and performance of the techniques.
- *Mobile Applications in Cervical cancer related Research*: There can be found numerous mobile apps for facilitating cervical cancer patients. These mobile apps are classified into three groups, namely: raising awareness, assessing cervical cancer risk, and providing healthcare information; under this analysis theme.

Each article was reviewed independently to extract the stated data. The findings were then aggregated, while any conflicts were resolved through discussion to avoid any possible biases. The extracted data was used to serve the purpose of evaluating, analyzing, and summarizing the existing research and is discussed in the next section.

## Results

### Research purposes and objectives

The objectives of the selected studies were categorized into four groups to highlight the key perspectives, covered in the domain of cervical cancer research including raising awareness, predicting cervical cancer, facilitating treatment and evaluating performance or feasibility (see Table 1).

**Table 1:** Key purposes of the reviewed studies.

Purposes	Brief Description	Reference	Frequency
Raising awareness	Developing a game-based learning tool that educates mobile technology users about cervical cancer	[28]	4
	Explore the association among knowledge, awareness, and practices regarding breast and cervical cancer among Turkish women in Gaziantep	[29]	
	Assessing the effect of education on health beliefs and practice of women	[30]	
	Determining the level of awareness of, and the uptake of cervical cancer screening	[31]	
Predicting cervical cancer	Developing a model with outlier detection and oversampling method.	[32]	12
	Finding the right method and building a mobile personal health record.	[33]	
	Developing mobile application for taking images, checking image focus, and identifying the precancerous lesions	[34]	
	Detecting cervical intraepithelial neoplasia of grade 2 or worse (CIN2+) as an adjunct to a conventional VIA and VILI, in comparison with detection by histopathologic examination.	[35]	
	Training supervised ML models and comparative evaluation of models	[36–38]	
	Establishing an ensemble prediction model based on the influencing factors for cervical cancer	[39]	
	Building A machine learning model to predict the recurrence- proneness for cervical cancer	[40]	
	Predicting the risk of cervical cancer by proposing a novel ensemble approach. A gene-assistance module is also included as an optional strategy to enhance the robustness of the prediction.	[41]	
	Developing three machine learning models such as Decision Tree, Random Forest, and XGBoost to predict cervical cancer from behavior and its variables and the research got significantly improved outcomes with 93.33% accuracy.	[42]	
	Predicting cervical cancer through different screening methods using data mining techniques like Boosted decision tree, decision forest and decision jungle algorithms	[43]	
Facilitating treatments	Developing a decision tree-based CDS system to integrate national guidelines to provide guidance to clinicians.	[44]	2
	Describing the implementation of VIA and Treat-immediate cryotherapy in the VIA positive cases-model	[45]	
Evaluating performance/ feasibility	Assessing the feasibility of a mobile health (m-Health) data collection system	[46]	8
	Evaluating the quality of smartphone images	[47]	
	Comparing cervical careHPV screening in a rural community	[48]	
	Assessing risks and highlighting to administrators who require the testing services using the mobile app	[49]	
	Examining women’s beliefs about causes of cervical cancer and reasons for undergoing a Pap smear	[50]	
	Evaluating the effectiveness of an educational intervention and assessing cervical cancer screening rates post-intervention	[51]	
	Reviewing existing experiences, achievements, constraints, and lessons learned in community-based, cervical cancer intervention programmes in developing countries	[52]	
	Testing the implementation of the novel job aid feature on the mobile colposcopy app during an EVA deployment in a cervical cancer screening camp	[53]	

The development of awareness is an instrumental factor for enhancing the treatment and prevention of cervical cancer. Recent studies showed that different educational interventions and methods for spreading awareness have significantly aided in improving the attitude of the general mass towards cervical cancer [54,55]. Moreover, research showed that fact based awareness messages from established organizations not only improves awareness among individuals but also causes further dissemination and spread of information through further sharing [55]. Furthermore, surveys show that despite having knowledge about cervical cancer treatment procedures, lack of awareness acts as a barrier towards attaining proper healthcare, especially in underdeveloped and developing countries [56,57]. Lack of awareness has been found to be responsible for the under-utilization of medical resources for cervical cancer in lower and middle income countries [57]. A total of four articles (15.38%) focused to raise awareness about cervical cancer, HPV, and the disease risks associated with HPV infection among lowincome countries. For example, Karadag et. al.[29] created a questionnaire to assess the relationship between breast and cervical cancer screening behaviors, and knowledge and awareness about cervical cancer among Turkish women in Gaziantep. They discovered that women had an insufficient understanding of breast and cervical cancer and that knowledge and behaviors improved with increasing education levels, implying the need for national and regional planning, implementation, and evaluation of health policies and healthcare services. In another study, Ruiz-López et al.[28] developed a smartphone-based game for raising awareness among mass people about the Human Papilloma Virus (HPV), which is a common cause of cervical cancer [58]. Shojaeizadeh et. al. [30] conducted research in order to understand the effect of educational programs on increasing cervical cancer screening behavior among women. In this research, questionnaires were used as a data collection tool. Upon the collection of data, the Wilcoxon test and Kruskal-Wallis test were performed on the data as well. The study revealed that using the Health Belief Model, significant enhancement of participants’ knowledge can be achieved, which can lead to better healthcare practices and improved attitudes towards cervical cancer. This study also revealed that for digital systems, the user interface should be designed in such a way that even an illiterate woman can understand and use it easily.

Twelve studies (46.15%) worked on cervical cancer prediction using machine learning and images. For instance, the study conducted by Badriyah et. al.[33] developed three machine learning models for cervical cancer classification including Logistic Regression, Naive Byes, and Decision Tree. The models were mainly using a set of hospital data which included 9 features (age, vaginal bleeding, discharge, smell, itching, abdomen pain, lump, pain while having sex, and other symptoms). While evaluating the machine learning models, it was found that logistic regression performed better than the other two methods in terms of accuracy, precision, and recall. Again, another study, driven by Ricard-Gauthier et al. [35], analyzed the use of Smartphones as Adjuvant Tools for Cervical Cancer screening in low-resource settings. Upon the completion of the analysis, the authors found the feasibility and reliability of smartphone images being used by the physicians for cervical cancer screening.

Providing proper treatment to cervical cancer patients immediately after the conduction of the screening methods can greatly diminish the impact of cervical cancer [59]. Research has shown that scaling up all treatment modalities could improve global net survival rate of cervical cancer patients [60]. In addition to expanding treatment, improving quality of care could raise survival rate significantly [60]. Telemedicine based remote treatment procedures have been found to aid in dealing with the medical resource constraints in Low and Middle Income Countries [61]. And research has shown that virtual and remote healthcare yields similar patient satisfaction when compared with in person healthcare practices for cancerous diseases [62]. With the increase in use of digital devices in lower and middle income countries [63], the adoption of telemedicine based approaches can further enhance the cervical cancer treatment strategies. Two studies (7.69%) discussed how the treatment of cervical cancer can be facilitated. Wagholikar et. al.[44] used a decision tree to create an automated clinical decision support (CDS) system, in which Natural Language Processing identified the parameters from cervical pathology and clinical notes. Again, Nuranna et. al. [45] conducted a systematic observational study in Jakarta for four years from 2007 to 2010. After evaluating and analyzing the data from the study, the authors determined that the Proactive-VO model is a suitable model for cervical cancer prevention. Though this research does not focus on developing any systems for cervical cancer screening, since the Proactive-VO model requires the healthcare practitioner to perform a visual inspection, this system can be further enhanced using image processing and machine learning techniques.

Eight studies (30.77%) evaluated the feasibility and performance of mobile health care service, images to detect cervical cancer and assessing risks to them who requires the testing services using mobile application. Quercia et. al.[46] undertook research to see if a mobile health (m-Health) data gathering system could be used to track women who were taking part in a cervical cancer screening program in Madagascar. The study confirmed that a smartphone m-Health application data collection system is the feasible and reliable for a cervical cancer screening campaign in an LMIC, and suggested that the creation of a computer-based patient record that is accessible to on- and off-site caregivers can improve the quality of care in cervical cancer screening, and that women’s follow-up is needed to confirm the efficiency on a long-term scale.Again, Labani et. al.[48] reviewed the HPV testing method to analyze its effectiveness and feasibility. Upon the completion of the analysis, it was found that HPV testing is superior to several other contemporary testing methods.

Since in this test, the detection is done by visually analyzing the collected cell samples, there are ample scopes to integrate machine learning and image processing techniques to further enhance the process. On the other hand, Munoz et. al.[49] created a mobile app for measuring knowledge, identifying dangers, and alerting administrators who require testing services.

It can be observed that recent research has emphasized the integration of artificial intelligence rather than conventional methods of detecting cervical cancer. As technology advances, more emphasis is placed on developing mobile applications in every aspect, including cervical cancer-related applications, thus, mobile applications are being developed to predict cervical cancer stage, raise awareness, and evaluate previous methods of detecting cervical cancer, such as VIA, screening methods, and so on.

In summary, it can be said that the most of the reviewed articles were focused on the technology either to raise awareness or to screen patients with cervical cancer. However, ML models are employed by few studies (n = 5), to determine the best ML-algorithm for predicting cervical cancer, identifying precancerous lesions from photographs, and providing physicians with guidelines and information.

### Publications profile

**Fig 3:**
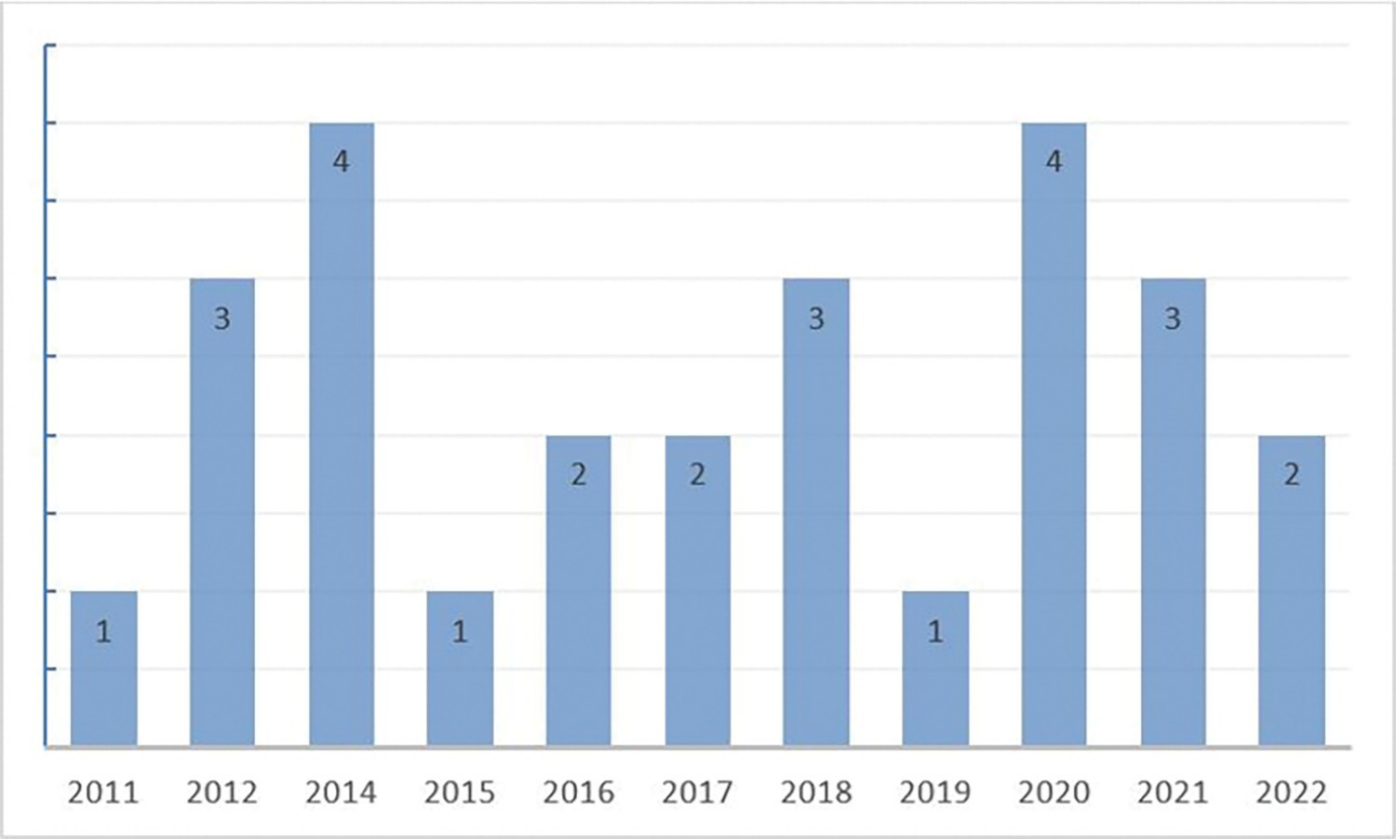
Publication year and number of publication.

The number of reviewed research articles, published each year between 2011 to 2022, are elucidated in Fig 3, in the form of a bar-chart. It can be seen from Fig 3, that the highest number (n = 2) of publications was in 2014 and 2020. Next, the second highest number of publications (n = 3), were found in the year 2012, 2018, and 2021, while no publications were found in the year 2013. However, in 2016, 2017 and 2022 two articles were published within our domain and the other years in the timeline had one publication in each year. Among the reviewed articles, 22 (84.62%) articles chosen were published in journals, while the remaining 4 (15.38%) were presented at conferences. The journal articles were from five different publishers, including IEEE, MDPI, ASCO, Dovepress, and Taylor and Francis. Two of the conference articles were published in ICAITI (International Conference on Applied Information Technology and Innovation) and ICIIC 2021 (3rd International Conference on Integrated Intelligent Computing Communication and Security). It can be observed that the majority of articles are published in medical-related publications; such as: JCO Oncology Practice, Health Care for Women International, International Journal of Women’s Health, and the likes, while few articles are published by non-medical journals, such as: JMIR serious games, and SENSORS. In brief, it is evident that the studies conducted in the field of cervical cancer research are commonly published in journals, rather than presented at conferences. Although a significant amount of research is seen on different journals and conferences, there is no dedicated journal in the field of cervical cancer research.

### Context of existing studies

Among the 26 articles, 11 (42.31%) articles were focused on a specific country context, which include Madagascar (n = 3), Iran (n = 2), Turkey (n = 1), Indonesia (n = 1), India (n = 1), Kenya (n = 1), Jamaica (n = 1), and Ethiopia (n = 1) while the rest of the articles were written for a wide or global audience (n = 7). For example: the study by Karadag et. al. [29] conducted in Gaziantep, a province in Turkey, assessed the relationship between breast and cervical cancer screening behaviors, and knowledge and awareness about cervical cancer. The study discovered that women had insufficient understanding about breast and cervical cancer, and that knowledge and behaviors improved with increasing education levels, implying the need for national and regional planning, implementation, and evaluation of health policies and healthcare services. Another study conducted in Jakarta, Indonesia; used a comprehensive strategy to explain the implementation of a single visit approach or see-visual inspection of the cervix with acetic acid (VIA) and Treat-immediate cryotherapy in VIA positive cases-model for cervical cancer prevention [45]. The Proactive-VO model was shown to be a potential strategy to screen and treat precancerous lesions in low-resource settings in this observational investigation. Nonetheless, one study conducted in Madagascar, investigated whether a mobile health (m- Health) data gathering system could be used to track women who were taking part in a cervical cancer screening program, confirming that a smartphone m-Health application data collection system is the feasible and reliable for a cervical cancer screening campaign in an LMIC, and suggested that the creation of a computer-based patient record that is accessible to on- and off-site caregivers can improve the quality of care in cervical cancer screening.

It can be observed that articles based on a contextual viewpoint, are mostly conducted in developing countries, primarily concerned with raising awareness and assessing knowledge of cervical cancer in these nations, as women in these countries are frequently denied access to education and consequently lack information about this type of lesion. However, these context-based research outcomes are not all generalizable because not all nations in the globe are low-resource or underdeveloped, hence the assessment procedures used in a developed country would provide radically different results. The global perspective publications have combined machine learning to forecast cancer stages because this sort of work contains enormous data on cancer.

### Exploring the AI techniques

A number of researches were conducted focusing on the development of machine learning-based systems for cervical cancer screening (see Table 2). The majority of the studies employed machine learning approaches are in the category of predicting the stage of cancer in its early stages. The other categories are Screening image evaluation and Automated recommendation. Ten articles focused on predicting cervical cancer in its early stages. The ML-based algorithms such as Naive Bayes, decision trees, and logistic regression are some of the most commonly utilized algorithms in the research studies. For example, Ijaz et. al.[32] developed a cervical cancer prediction model based on DBSCAN and iForest. The authors used SMOTE and SMOTE Tomek methods for data augmentation. Only a single dataset from which the feature identification was done while the model was evaluated using several metrics including precision, recall, f1-score, and specificity. However, in this study, the authors only used. As per the plans for their future work, the inclusion of more datasets will not only improve the performance of the model but also may result in the selection of new features. Again, Naveen et al. [36] developed a Decision Tree-based to detect the early stage of Cervical Cancer. The system was evaluated against the Multilayer Perceptron and the Naive Bayes model in terms of several metrics including Kappa Statistics, Mean Absolute Error, False Negative Rate, False Positive Rate, True Negative Rate, and True Positive Rate and Confusion Matrix. Finally, a mobile app was developed where the model was deployed.

**Table 2:** A summary of the algorithm used in the literature for different objectives.

Objectives	Algorithms	Evaluation Results	Literature
Disease prediction	SVM, MLP, Logistic Regression, Naïve Bayes, KNN, Random Forest	Best performance achieved by Random Forrest with noise (DBSCAN) and SMOTE for Biopsy. Precision- 97.025%, Recall- 98.039%, Specificity- 95.939%, F1 Score- 97.006%, Accuracy 97.007%.	[32]
	Decision Tree, Multilayer Perceptron and Naive Bayes	Best result found by C4.5 (J48) Decision Tree with an accuracy of 94.18%	[36]
	Logistic Regression, Naïve Bayes and Decision Trees	Logistic Regression method used with a high accuracy level of 95.12%	[33]
	SVM, C&R Tree, QUEST, RBF, MLP	Decision tree to be the best predictor	[37]
	Logistic Regression, Decision Tree, Random Forest, Ensemble Methods including bagging, boosting and voting	Best accuracy was gained by SMOTEVoting-PCA with 97.44% accuracy.	[38]
	Logistic Regression, Decision Tree Classifier, Naïve Bayes Classifier, Random Forest Classifier, K-Nearest Neighbor, Support Vector Machine and Deep Neural Networks	Voting Classifier had the highest accuracy (97–99) than DNN classifier	[39]
	SVM, C5.0, Extreme learning machine (ELM)	C5.0 model generated the best classification result with average correct classification rate of 96.0%.	[40]
	Logistic Regression, Decision Tree, Super Vector Machine, Multilayer Perceptron, K-Nearest Neighbors, An Ensemble strategy	Their proposed ensemble method ranks best in accuracy with 83.16%.	[41]
	Decision Tree Classifier, Random Forest Classifier, XGBoost Classifier	The accuracy of their proposed models of the decision tree, random forest, and XGBoost classifier found was 93.33%	[42]
	Boosted decision tree, decision forest and decision jungle	Boosted decision tree provided a very high prediction with 0.978 on the AUROC curve.	[43]
Screening Image Evaluation	Automated Visual Evaluation (AVE)	Area under the ROC curve (AUC) of 0.95	[34]
Automated Recommendation	Decision Tree, NLP	Overall accuracy of 87% (Decision Tree), NLP to determine parameters from cervical pathology and clinical notes	[44]

In another study [34], author focused to the screening image evaluation where they designed and developed a deep Learning based image evaluation system for cervical pre- screening using smartphones. The model was trained using transfer learning where MSCOCO dataset weights were used. Finally, the model was evaluated using the metrics of false-positive and time duration. Authors found the area under the ROC curve to be 0.95. One article was found in the category of automated recommendation. Such as, Wagholikar et al. [44] developed an automated recommendation system for cervical cancer screening and surveillance purposes. The system was developed using Decision Tree which was trained based on the data from the national guidelines used by clinicians for cervical cancer screening. The system was evaluated on 333 patients wherein 87% of the cases, the system was successful.

### Mobile applications in cervical cancer

A number of cervical cancer related applications are developed in the past year for serving different purposes to different people. The developed mobile applications are categorized into three groups: raising awareness, assessing cervical cancer risk, and providing healthcare pieces of information (see Table 3). Seven applications are found to be developed to raise awareness and spread education on cervical cancer. For example, the app “Cervical Cancer” by Fumo Medical shares medical knowledge about cervical cancer among women who use mobile phones. It spreads awareness by containing information about the cervix (cervix anatomy, cervix conditions, cervix tests, cervix treatments), Pathophysiology (all information about HPV infections), Etiology and Epidemiology, Clinical Presentation (information about history, physical examination), Symptoms and Cause (information about symptoms and cause), Diagnosis (information about cervical cancer types and diagnosis), Treatment (information about various kinds of treatment like surgery, radiation, chemotherapy, immunotherapy, supportive care), Complication of cervical cancer and Prevention. Again, “Cervical Cancer”by Nature Healthy Care is a forum that raises awareness by providing cervical cancer-related articles to the user and it collects new articles every day to keep the users updated. One app found to Assessing cervical cancer risk. The application named Cervical Cancer Risk Assessment tool assesses cervical cancer risk by containing information and questionnaire about cervical cancer symptoms. An app is found to provide health care information named "Cervical Cancer Tracker" that uses GPS to search nearby hospitals, store Pap smear test info and present a calendar option for setting events.

**Table 3:** A summary of the mobile applications on cervical cancer for different objectives.

Purposes	Application Name	Brief Description	Features	Platform	User	Reference	Frequency
Raising awareness	Cervical Cancer by Fumo Medical	Sharing medical knowledge about cervical cancer	Contains information about the cervix, pathophysio- logy, etiology and epidemiology, clinical presentation, symptoms and cause, diagnosis, treatment, complica- tion of cervical cancer and prevention	Android	Doctors, nurses, patients, general people	[64]	7
	Cervical Cancer by Natural Healthy Care	Spreading awareness about cervical cancer and provide knowledge related to treatment	Contains general information and guidelines about cervical cancer	Android	Patients	[65]	
	Cervical Cancer Guide	Spreading awareness about cervical cancer and provide knowledge related to treatment through video contents	Contains information about cervical cancer’s symptoms and cause, treatment and video lessons	Android	Patients, general People	[66]	
	Cervical Cancer Forum	Conducting discussions related to cervical cancer	Intiates discussions where users can take part in	Android	Patients, general People	[67]	
	Cervical Cancer Personal Remedies	Ensuring cervical cancer prevention through healthier lifestyle and diet.	Contains nutrition guidelines for cervical cancer and guidelines for additional diseases (depression, obesity, high blood pressure, high cholesterol, stress, and vitamin D deficiency)	Android	General people, patients	[68]	
	Cervical Cancer- News	Providing cervical cancer related articles	Provides a list of online articles related to cervical cancer and collects new articles everyday	Android	General people, patients	[69]	
	Cervical Cancer Tracker	Managing cervical cancer information	Provides basic information about cervical cancer (description, causes, prevention, pap smear, special population) and uses GPS to search nearby hospitals	Android	General people, patients, doctors	[70]	
Assessing cervical cancer risk	Cervical Cancer Risk Assessment tool	Assessing cervical cancer symptoms using questionnaire	Contains information and questionnaire about cervical cancer symptoms	Android	General people, patients	[71]	1
Providing health care information	Cervical Cancer Tracker	Using GPS to search nearby hospitals and storing pap smear test information	Uses GPS by which nearby hospitals can be searched, Pap smear test info can be stored and calendar option is present for setting events.	Android	General people, patients, doctors	[70]	1

Again, due to the increased accessibility of smartphone devices, a number of studies were conducted out focusing on the development of mobile applications for aiding in the prevention and screening of cervical cancer and spreading awareness among the general mass. The articles found to work on mobile applications are shown in Table 4. Zuluaga et. al.[49] developed a mobile app for spreading cervical and breast has cancer symptoms. If the probability of having cancer was detected, a dedicated team was informed by the app to contact the woman and provide the necessary support. This study determined that IT cost is a significant aspect of such healthcare apps and the involvement of healthcare facilities is required immensely for its smooth functioning. This research had some limitations because the developed process made use of intense manual labor and not much use of automation was done to remedy it. Quercia et al.[46] developed a mobile-based application, to help in providing cervical cancer screening services. A total of 151 women were recruited to evaluate the system. The authors also rigorously evaluated the system to identify different technical problems and later fixed the feasibility and reliability of a smartphone m-Health application data collection system for a cervical cancer screening campaign in a lower or middle-income country. Gallay et al.[47], in their work, proposed and developed a smartphone image application to serve as an alternative to the colposcopy method. The application was de- signed to obtain high-resolution images of the cervix. Furthermore, a database was de- signed to efficiently store the patient’s information along with the patient’s data. A qualitative evaluation of the system was done where it was found that in 93.3% of the cases, the app was useful for the healthcare providers for performing accurate diagnoses. How- ever, in their work, no measure was taken to automate the diagnosis process without requiring any human intervention. Ruiz et al.[28] designed, developed, and evaluated a mobile game to raise awareness about the Human Papilloma Virus and motivate people to take the necessary precautionary measures. The research further showed a significant correlation between awareness and precautionary measures against cervical cancers.

**Table 4:** A summary of the studies focused to develop mobile application for different objectives.

Purposes	Brief Description	Method	Context	Ref	Freq
Predicting cervical cancer	Developing a cervical cancer prediction model with outlier detection and oversampling method.	Prediction model consists of outlier detection based on DBSCAN and iForest, and SMOTE, and SMOTETomek to balance the data with RF for cancer prediction	Global	[32]	2
	Finding the right method for predicting cervical cancer and building a mobile personal health record related to the prediction and treatment of cervical cancer disease.	Collection of dataset from a hospital with 9 input features, trained three models which are based on Logistic Regression, Naive Byes, Decision Tree	Global	[44]	
Raising awareness	Developing a mobile app for knowledge evaluation, assessing risks, and highlighting to administrators who require the testing services.	Preparation of questionnaires and modifications of the questionnaires via feedback from app	Global	[49]	1
Evaluating experience	Assessing women experience with m-health cervical cancer screening	Women screened via HPV self-collection, 120 women were provided the Patient Experience Questionnaire about their visit and perceptions and attitudes.	Rwanda	[72]	1
Collecting data	Assessing the feasibility of a mobile health (m-Health) data collection system to facilitate monitoring of women participating in cervical cancer screening campaigns.	Registration of clinical data, which is then transmitted onto a secure, Web-based platform through internet connection	Madagascar	[47]	1

### Topical associations

**Fig 4:**
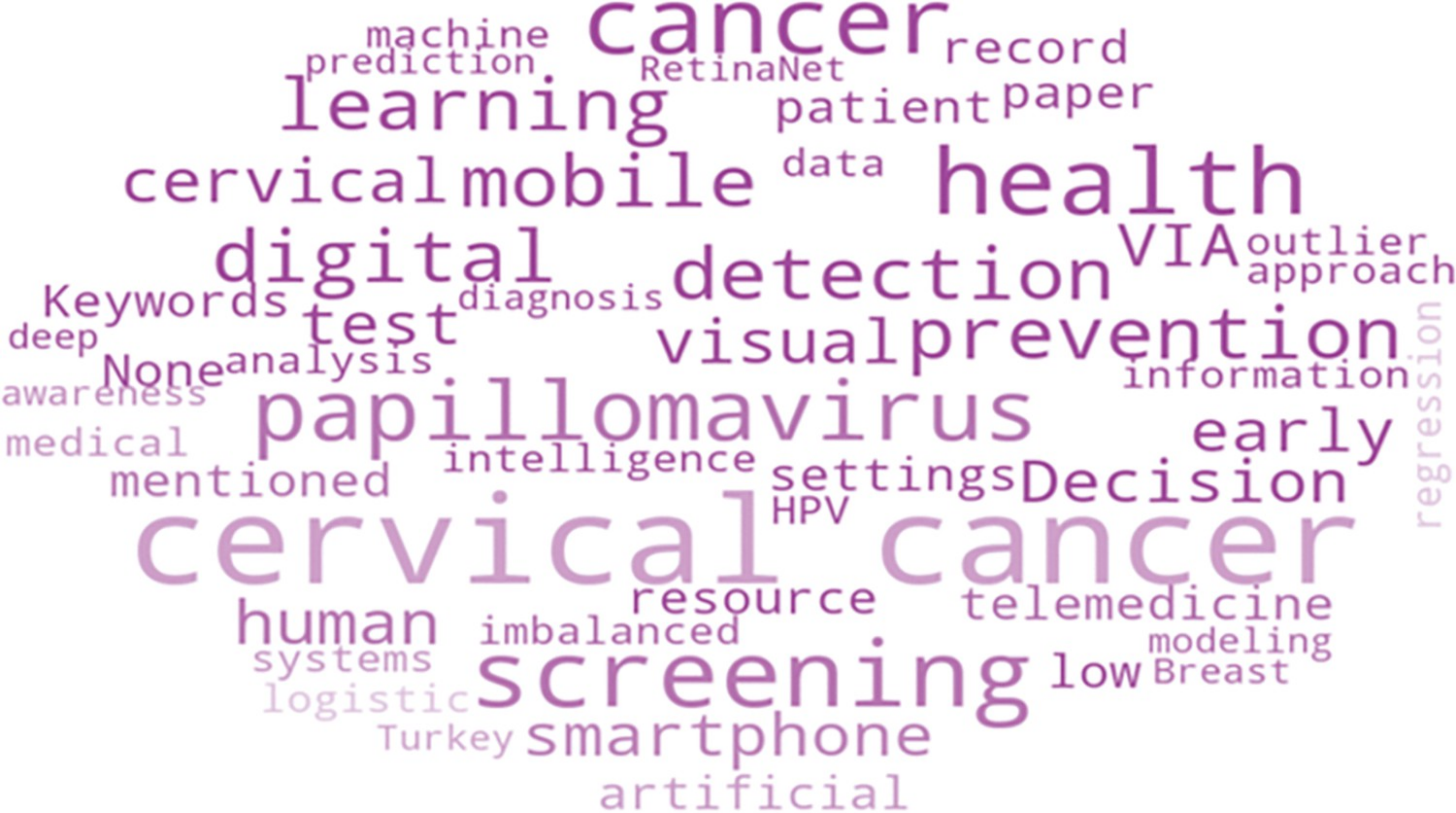
Word cloud based on the keywords.

Fig 4 shows the terms that appeared as the keywords on the reviewed articles, in the form of a word cloud. Generally, word clouds are used to represent the effect factor of a word in a particular context, where the size of a word in a word cloud depicts its frequency of presence in the context, therefore the larger a word in the word cloud is, the more it appears. Cervical, cancer, screening, papillomavirus, prevention, mobile, health and VIA are the most commonly used keywords in this context. The core focus of all of these articles is cervical cancer, and human papillomavirus is one of the primary causes of cervical cancer, according to the word cloud of keywords. The VIA method is used to determine the existence of cervical cancer. Other small terms indicate that work on mobile apps is underway. In addition, a number of machine learning methods are employed and few works additionally contain image processing.

**Fig 5:**
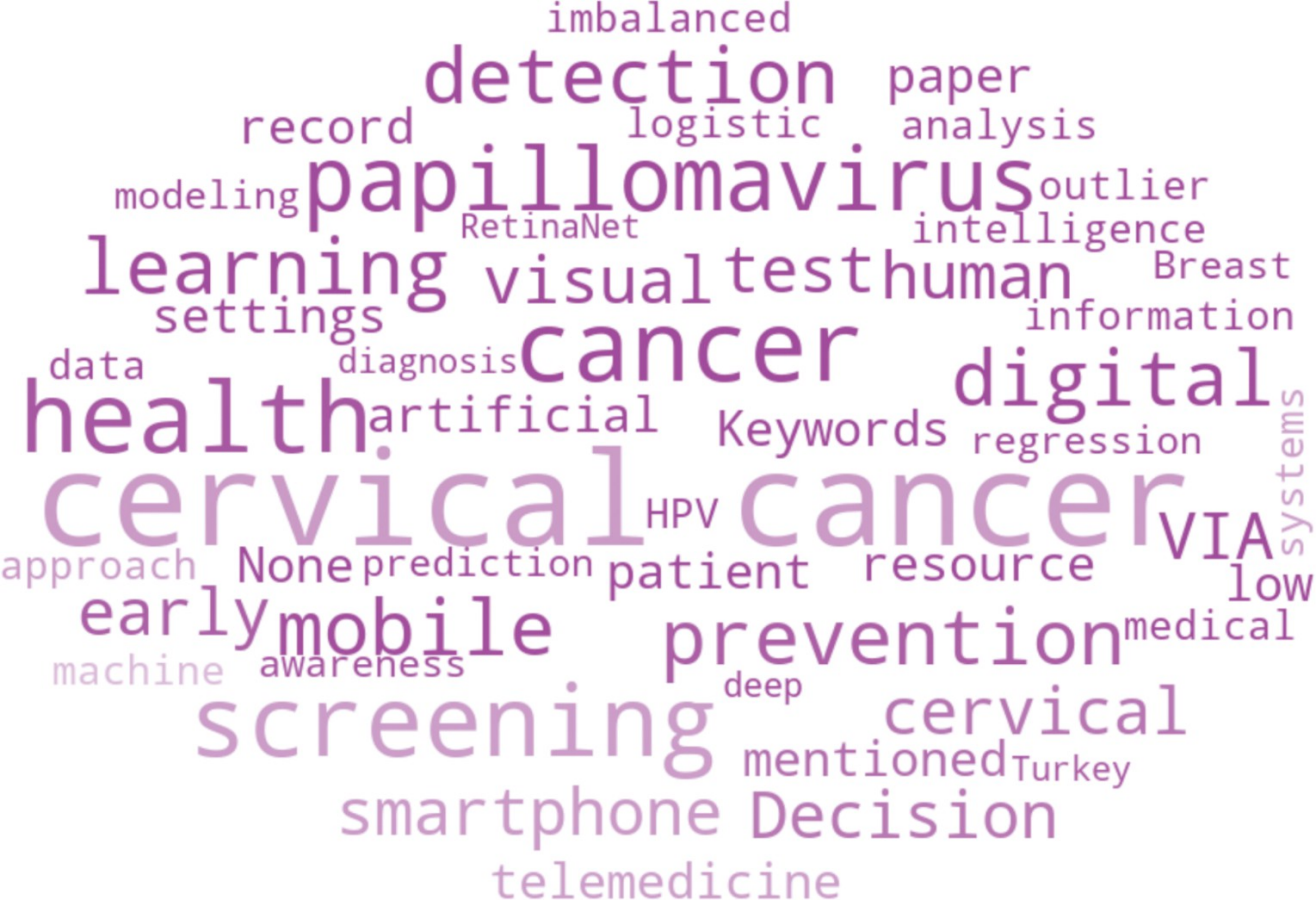
Word cloud based on the articles’ titles.

The most prevalent words in the titles of chosen papers, as shown in Fig 5, are cervical, cancer, papillomavirus, screening, and health. Other minor phrases like mobile, digital, modeling and artificial offer an understanding of the work that has gone into developing mobile apps or digital techniques to detect cervical cancer. Prediction, early stage, risk assessment, and cervical precancer screening are all phrases that suggest that the majority of effort is done to discover cervical cancer in its early stages, which protects women from developing severe forms of the illness.

## Discussions

### Findings from the analysis

#### Research purposes and objectives

The research purposes of the selected articles mostly focused on predicting cervical cancer disease using Artificial Intelligence (AI). Few articles focused on evaluating the existing methods of screening cervical cancer and the feasibility of existing mobile apps, while some focused on the evaluation of images taken by mobile apps for getting treatment by a doctor. Other articles focused on facilitating treatment for women and raising awareness on this topic.

#### Publication type and year

All the articles were published in journals and conferences. The articles in this section are published more recently focusing on technologies. Whereas recent research is focused on the technological aspect of this sector such as using AI techniques to predict disease, remote treatment using mobile images, and the like, the earlier research works were mostly focused on screening methods, knowledge evaluation, and spreading awareness.

#### Context of existing studies

Most of the research was done in low or lower-middle-income countries such as India, Madagascar, and Kenya. There were very few studies found that were done in upper income or developed countries. Research works were done in the global context as well, which shows that the low or lower-middle-class countries are still backward in the field of knowledge and awareness of cervical cancer.

#### Exploring the AI techniques

The articles created in the context of cervical cancer were focusing on the prediction of cancer disease. The research used machine learning models to predict cervical cancer. AI and machine learning techniques are primarily used for predicting cervical cancer and exploring the best predictors by finding out the maximum accuracy of the models. Some articles worked on the evaluation of screening images and found the best predictive model for better images. Few articles were on automated recommendation for doctors using natural language processing.

#### Mobile applications in cervical cancer

A lot of mobile applications were developed focusing on cervical cancer. Most applications were made to raise awareness- such as the reasons, results, and symptoms of cervical cancer. Applications were also developed to suggest doctors and nearby hospitals to the user for screening purposes. Very few applications are found that were developed to predict disease (cervical cancer) by using machine learning or deep learning techniques.

#### Topical association

The impact of the relationships between the topics of the chosen articles is shown by the word cloud created using the keywords and titles. The most used words for both titles and keywords include papillomavirus, cervical cancer, screening, health, and cancer. This demonstrates that the publications included in the review are closely related and the majority of them were done in the setting of ‘cervical cancer’.

### Future research agenda

#### Exploring the innovative approaches for spreading awareness

Exploring the innovative approaches for spreading awareness The findings from the data analysis show that very few researches have been conducted in raising awareness (n = 4), despite the fact that development of awareness has been found to be a key factor in diminishing the impact of cervical cancer [54,55]. The data analysis results have shown that researchers have strived to develop awareness oriented measures through several approaches including development of game based learning tool [28], conducting exploratory study to find further association between knowledge, education and awareness with cervical cancer related practices [29,30]. However, these measures are not sufficient and there are more avenues that are required to be explored in this regard. For example, research has identified that the use of animated cartoons can play a significant role in healthcare awareness [73]. Thus the impact of animated cartoons in cervical cancer can be determined through further studies. Similarly, the social media has been found to play a key role in spreading health related awareness [74]. Thus, the role of social media can also be explored for diminishing the severity of cervical cancer. Moreover, most of the exploratory researches were conducted in developed countries [28–31]. As such, there are potential scopes for conducting future studies in the context of developing and under developed countries where several barriers are existing in healthcare including superstitions, religious fanaticism, patriarchy, etc [75].

#### Facilitating treatment through the uses of emerging technologies

In order to cure cervical cancer, very few studies have been carried out on implementing or analysing different state of the art measures. For example, Nuranna et al. [45] described the implementation of visual inspection of the cervix with acetic acid (VIA)-and treatment with cryotherapy, while Wagholikar et al. [44] developed a decision tree based clinical support system following the national guidelines. However, the number of researches in this regard clearly indicate that more studies can be conducted to identify further state of the art techniques. Moreover, there are some established practices that facilitate healthcare which can be extended for cervical cancer for research purposes. For example, research has shown that regular checkups can act as means of prevention for different cancerous diseases [76]. This avenue can be further explored for cervical cancer through longitudinal studies and different applications can be implemented through emerging technologies for ensuring regular check-ups and advanced treatment. Since, the emerging technologies (e.g., artificial intelligence, Internet of Things, next generation remote health care, etc.) may create a promising platform for facilitating novel treatment options for the cervical cancer patients. Moreover, research has also found that knowledge sharing forums under consistent monitoring by authoritative figures can play a role in guiding people towards attaining the proper treatment facilities [77]. This can be another case which can be associated with cervical cancer while conducting research.

#### Pursue research considering different study context

The findings from data analysis show that a considerable number of researches were conducted considering both the context of particular countries (52.33%) and the global context (46.67%). However, the analysis of the articles, which was based on the context of specific countries, revealed that most of the research was conducted on developed/developing countries (Turkey, Indonesia, India) where patriarchy is prevalent to some extent [78]. Furthermore, the studies were primarily concerned with raising awareness and assessing knowledge of cervical cancer in these nations, as women in these countries are frequently denied access to education and consequently lack information about this type of lesion. Thus a significant research gap is observed in this regard, because around the globe, the percentage of underdeveloped countries is more [79]. Also the demographics vary vastly among these countries. When considering the condition of underdeveloped countries, different other demographics are to be considered including education, access to healthcare, religious superstitions, etc [80]. Thus more research works can be proposed integrating the demographics information of the underdeveloped countries. Similarly, other than focusing only towards raising awareness and assessing knowledge, more contextual research objectives like evaluation of treatment facilities, studying the broad impact of culture, analyzing the trend of change in attitude towards cervical cancer with the passage of time, etc. can be explored in the future work. Furthermore, it is observed that there has been a limited number of clinical studies conducted. Further investigations using clinical-based data could yield more authentic results and potentially revolutionize understanding of cervical cancer. This suggests an area of opportunity for future research in the field.

#### Explore more features for machine learning based researches

In the case of machine learning based prediction approaches, the data analysis in this research showed that very few machine learning features were explored for the research. One of the researchers considered only 9 features [33] for developing 3 machine learning models while another research made use of normal image pixels for diagnosis [35]. However, more research can be conducting for analyzing feature selection or optimization. Firstly, there are opportunities for experimenting with more features since in open source repositories, datasets consisting of several features are available. For example, in [81], there are about 35 features for cervical cancer detection. Secondly, for the nonimage based features, there can be research which can result in determining the optimal features using different feature selection methods [82]. Such exploration regarding the dataset features may result in even better and effective models. Thirdly, there are also ample opportunities for finding out new features from different processes [83] including expert consultation, interviewing stakeholders and scientific article review. Finally, there can be more studies were different image modalities can be considered [84] where different imaging techniques other than using normal camera based images can be explored for cervical cancer detection, for example CT scan images, MRI images, etc.

#### Inclusion of more machine learning based studies

The data analysis in this study showed that a number of machine learning algorithms were incorporated for cervical cancer screening purposes [32–34,36,44]. Nevertheless, very few machine learning algorithms have been used for this purpose. Some state of the art Machine learning algorithms is yet to be explored in this regard. For example, Neural Networks, boosting algorithms, Federated Learning, etc which are found to perform better when trained and tuned properly [85,86]. These research gaps can be utilised to create novel research agendas and thus further improve the screening processes for cervical cancer.

#### Use of data from different stages of cervical cancer

The analysis results showed that most of the researchers were concerned about the early stages of cervical cancer [32–34,36,44]. Though this is a praiseworthy approach which can aid in early prevention of cervical cancer and its associated complications, there are necessities for conducting research with data from the later stages of the cancer as well. Again recent studies have shown that machine learning based approaches can play a role in decision making in the later stages of cancerous diseases [87]. Potential future research focusing towards the later stages of cervical cancer will not only aid in limiting the existing research gap but also be instrumental in lowering the mortality rate due to cervical cancer.

#### Further development of mobile applications

This research has identified a number of mobile applications developed in the context of dealing with cervical cancer (see Table 3 for reference). Though the applications were found to be functional in fulfilling the objectives of the application developers, there are some major concerns related to the apps. Most of the apps developed for dealing with cervical cancer were used for the purpose of spreading awareness (77.77%), but only two were found to aid in facilitating treatment (22.22%) out of which only (11.11%) of the apps were dedicated towards providing screening services. Thus more research and investment can be directed towards developing apps for facilitating treatment and enhancing screening services Furthermore, none of the mobile applications were found to take into consideration the application design principles from the perspective of human computer interaction principles. The usability of mobile apps varies based on the demographic characteristics of a person for example education level, culture, etc. [88]. Thus future research can be designed taking into consideration the design issues to enhance the usability and user experience.

#### Advancing usability and patient-centric design in cervical cancer mobile applications

The trajectory of future research in cervical cancer studies involves a concentrated effort towards propelling the development of mobile applications with a deliberate focus on enhancing usability and embracing a patient-centric design paradigm. The overarching objective is to establish a human-centric system, ensuring user-friendliness, especially for diverse demographics such as individuals who are illiterate, reside in rural areas, face economic barriers, or exist below the poverty line—cohorts often hesitant to engage in conventional medical consultations. Despite the proliferation of applications from varied perspectives, a discernible gap persists in specific domains such as usability, user experience (UX), human-computer interaction (HCI), and patient-centric design. Future research endeavors to systematically address this void by assessing the usability and user experience of digital solutions developed for the cervical cancer care and by integrating design principles that intricately consider the unique needs of users, which in turn will herald a new era of inclusive and effective mobile health applications.

### Conclusions

This article reviewed a number of articles that focused on cervical cancer. The publications were chosen from a variety of sources based on their relevance to the fields of raising awareness, facilitating treatment, and applying artificial intelligence to forecast cervical cancer disease.

This study outlines the areas that have been looked into in regards to cervical cancer as well as the objectives of the studies. The study also identifies any gaps in knowledge and areas where more research is needed in the domain of cervical cancer. For instance, there have been numerous studies conducted in the area of screening techniques. However, very few studies have focused on technology advancements in this area, such as exploring artificial intelligence and improving treatment. In order to cure cervical cancer as early as possible and lessen the impact it has on the women in our society, these fields need to be improved in the future. Consequently, our study provides direction for future work for both the researchers and to lessen the influence it can have on society.

The choice of keywords for this study has limited the number of publications that could be searched, however this limitation could be overcome if we continue to work on the study in the future. The fact that there are just a small number of the mobile applications assessed and that all of them solely concentrate on raising awareness while others do concentrate on facilitating treatments demonstrates how urgently this area needs investigation in order to better serve women. The overall number of reviewed publications was not very high because many more papers may have been removed for sharing similarities with the selected articles. Additionally, the absence of dataset in the field of cervical cancer prevented Artificial Intelligence from exploring.

This analysis thus points in the direction of potential future research in the subject of cervical cancer, which will benefit many women worldwide and decrease its effects. It also shows that Artificial Intelligence and Machine Learning is yet to be explored deeply, which could have a significant impact on our society.

## Supporting information

S1 Checklist PRISMA 2020 checklist. (DOCX)

## References

[1] Cervical cancer—statistics (2022). https://www.cancer.net/cancer-types/cervical-cancer/statistics.

[2] Yang EJ, Quick MC, Hanamornroongruang S, Lai K, Doyle LA, McKeon FD, et al. Microanatomy of the cervical and anorectal squamocolumnar junctions: a proposed model for anatomical differences in HPV-related cancer risk. Modern Pathology. 2015 Jul;28(7):994–1000. doi: 10.1038/modpathol.2015.54 25975286 PMC4490106

[3] Wright T.C. Jr, Kuhn L.: Alternative approaches to cervical cancer screening for developing countries. Best practice & research Clinical obstetrics & gynaecology 26(2), 197–208 (2012). doi: 10.1016/j.bpobgyn.2011.11.004 22385539

[4] Small W. Jr, Bacon M.A., Bajaj A., Chuang L.T., Fisher B.J., Harkenrider M.M., et al.: Cervical cancer: a global health crisis. Cancer 123(13), 2404–2412 (2017). doi: 10.1002/cncr.30667 28464289

[5] Kessler T.A.: Cervical cancer: prevention and early detection. In: Seminars in Oncology Nursing, vol. 33, pp. 172–183 (2017). Elsevier. 28343836 10.1016/j.soncn.2017.02.005

[6] Cervical cancer. World Health Organization: Geneva, 2018. WHO. Last accessed: 10 May, 2022.

[7] Kashyap N., Krishnan N., Kaur S., Ghai S.: Risk factors of cervical cancer: a case-control study. Asia-Pacific journal of oncology nursing 6(3), 308–314 (2019). doi: 10.4103/apjon.apjon_73_18 31259228 PMC6518992

[8] Schiffman M.H., Brinton L.A.: The epidemiology of cervical carcinogenesis. Cancer 76(S10), 1888–1901 (1995). doi: 10.1002/1097-0142(19951115)76:10+&lt;1888::aid-cncr2820761305&gt;3.0.co;2-h 8634980

[9] Mukakalisa I., Bindler R., Allen C., Dotson J.: Cervical cancer in developing countries: effective screening and preventive strategies with an application in rwanda. Health care for women international 35(7–9), 1065–1080 (2014). doi: 10.1080/07399332.2014.909433 24750113

[10] Kashyap N., Krishnan N., Kaur S., Ghai S.: Risk factors of cervical cancer: a case-control study. Asia-Pacific journal of oncology nursing 6(3), 308–314 (2019). doi: 10.4103/apjon.apjon_73_18 31259228 PMC6518992

[11] Wit E.M., Horenblas S.: Urological complications after treatment of cervical cancer. Nature reviews Urology 11(2), 110–117 (2014). doi: 10.1038/nrurol.2013.323 24473416

[12] Vermeer W.M., Bakker R.M., Kenter G.G., Stiggelbout A.M., Ter Kuile M.M.: Cervical cancer survivors’ and partners’ experiences with sexual dysfunction and psychosexual support. Supportive Care in Cancer 24(4), 1679–1687 (2016). doi: 10.1007/s00520-015-2925-0 26412245 PMC4766206

[13] Canfell K.: Towards the global elimination of cervical cancer. Papillomavirus research 8, 100170 (2019). doi: 10.1016/j.pvr.2019.100170 31176807 PMC6722296

[14] Brisson M., Kim J.J., Canfell K., Drolet M., Gingras G., Burger E.A., et al.: Impact of hpv vaccination and cervical screening on cervical cancer elimination: a comparative modelling analysis in 78 low-income and lower-middle-income countries. The Lancet 395(10224), 575–590 (2020). doi: 10.1016/S0140-6736(20)30068-4 32007141 PMC7043009

[15] Naz M.S.G., Kariman N., Ebadi A., Ozgoli G., Ghasemi V., Fakari F.R.: Educational interventions for cervical cancer screening behavior of women: a systematic review. Asian Pacific journal of cancer prevention: APJCP 19(4), 875 (2018). 29693331 10.22034/APJCP.2018.19.4.875 PMC6031778

[16] Singh S., Fletcher G.G., Yao X., Sussman J.: Virtual care in patients with cancer: a systematic review. Current Oncology 28(5), 3488–3506 (2021). doi: 10.3390/curroncol28050301 34590602 PMC8482228

[17] Rodriguez-Villa E., Naslund J., Keshavan M., Patel V., Torous J.: Making mental health more accessible in light of covid-19: Scalable digital health with digital navigators in low and middle-income countries. Asian journal of psychiatry 54, 102433 (2020). doi: 10.1016/j.ajp.2020.102433 33271713 PMC11694478

[18] Ponniah H.S., Shah V., Rad A.A., Vardanyan R., Miller G., Malawana J.: Theatres without borders: a systematic review of the use of intraoperative telemedicine in low-and middle-income countries (lmics). BMJ Innovations 7(4) (2021).

[19] Palmatier R.W., Houston M.B., Hulland J.: Review articles: Purpose, process, and structure. Springer (2018).

[20] Xue P., Wang J., Qin D., Yan H., Qu Y., Seery S., et al: Deep learning in image-based breast and cervical cancer detection: a systematic review and meta-analysis. NPJ digital medicine 5(1), 1–15 (2022). 35169217 10.1038/s41746-022-00559-z PMC8847584

[21] Peirson L., Fitzpatrick-Lewis D., Ciliska D., Warren R.: Screening for cervical cancer: a systematic review and meta-analysis. Systematic reviews 2(1), 1–14 (2013). doi: 10.1186/2046-4053-2-35 23706117 PMC3681632

[22] Lee Y.-H., Huang L.-H., Chen S.-H., Shao J.-H., Lai C.-H., Yang N.-P.: Effects of mobile application program (app)-assisted health education on preventive behaviors and cancer literacy among women with cervical intraepithelial neoplasia. International journal of environmental research and public health 18(21), 11603 (2021). doi: 10.3390/ijerph182111603 34770117 PMC8582743

[23] Rossman A.H., Reid H.W., Pieters M.M., Mizelle C., von Isenburg M., Ramanujam N., et al: Digital health strategies for cervical cancer control in low-and middle-income countries: systematic review of current implementations and gaps in research. Journal of medical Internet research 23(5), 23350 (2021). doi: 10.2196/23350 34042592 PMC8193495

[24] Shanthi P., Hareesha K., Kudva R.: Automated detection and classification of cervical cancer using pap smear microscopic images: a comprehensive review and future perspectives. Engineered Science 19, 20–41 (2022).

[25] Bhochhibhoya S., Dobbs P.D., Maness S.B.: Interventions using mhealth strategies to improve screening rates of cervical cancer: A scoping review. Preventive Medicine 143, 106387 (2021). doi: 10.1016/j.ypmed.2020.106387 33383069

[26] Champin D., Ramírez-Soto M.C., Vargas-Herrera J.: Use of smartphones for the detection of uterine cervical cancer: A systematic review. Cancers 13(23), 6047 (2021) doi: 10.3390/cancers13236047 34885157 PMC8656777

[27] Bedell S.L., Goldstein L.S., Goldstein A.R., Goldstein A.T.: Cervical cancer screening: past, present, and future. Sexual medicine reviews 8(1), 28–37 (2020). doi: 10.1016/j.sxmr.2019.09.005 31791846

[28] Ruiz-López T., Sen S., Jakobsen E., Tropé A., Castle P.E., Hansen B.T., et al: Fighthpv: design and evaluation of a mobile game to raise awareness about human papillomavirus and nudge people to take action against cervical cancer. JMIR serious games 7(2), 8540 (2019). doi: 10.2196/games.8540 30958271 PMC6475825

[29] Karadag G., Gungormus Z., Surucu R., Savas E., Bicer F.: Awareness and practices regarding breast and cervical cancer among turkish women in gazientep. Asian Pacific Journal of Cancer Prevention 15(3), 1093–1098 (2014). doi: 10.7314/apjcp.2014.15.3.1093 24606424

[30] Shojaeizadeh D., Hashemi S.Z., Moeini B., Poorolajal J.: The effect of educational program on increasing cervical cancer screening behavior among women in hamadan, iran: Applying health belief model. Journal of Research in Health Sciences 1(1), 20–25 (2011). 22911943

[31] Erku D.A., Netere A.K., Mersha A.G., Abebe S.A., Mekuria A.B., Belachew S.A.: Comprehensive knowledge and uptake of cervical cancer screening is low among women living with hiv/aids in northwest ethiopia. Gynecologic oncology research and practice 4(1), 1–7 (2017). doi: 10.1186/s40661-017-0057-6 29276611 PMC5738137

[32] Ijaz M.F., Attique M., Son Y.: Data-driven cervical cancer prediction model with outlier detection and over-sampling methods. Sensors 20(10), 2809 (2020). doi: 10.3390/s20102809 32429090 PMC7284557

[33] Badriyah T., Ratudduja I., Desy I.P., Syarif I.: Assessing risk prediction of cervical cancer in mobile personal health records (mphr). In: 2018 International Conference on Applied Information Technology and Innovation (ICAITI), pp. 170–175 (2018). IEEE.

[34] Hu L., Horning M.P., Banik D., Ajenifuja O.K., Adepiti C.A., Yeates K., et al: Deep learning-based image evaluation for cervical precancer screening with a smartphone targeting low resource settings–engineering approach. In: 2020 42nd Annual International Conference of the IEEE Engineering in Medicine & Biology Society (EMBC), pp. 1944–1949 (2020). IEEE. 10.1109/EMBC44109.2020.9175863 33018383

[35] Ricard-Gauthier D., Wisniak A., Catarino R., van Rossum A.F., Meyer-Hamme U., Negulescu R., et al: Use of smartphones as adjuvant tools for cervical cancer screening in low-resource settings. Journal of lower genital tract disease 19(4), 295–300 (2015). doi: 10.1097/LGT.0000000000000136 26247260

[36] Naveen R.C., Asha K., Prasad G.K., Manjula G.: Women healthcare mobile app-an approach to predict early stage of cervical cancer. In: 3rd International Conference on Integrated Intelligent Computing Communication & Security (ICIIC 2021), pp. 175–182 (2021). Atlantis Press.

[37] Asadi F., Salehnasab C., Ajori L.: Supervised algorithms of machine learning for the prediction of cervical cancer. Journal of biomedical physics & engineering 10(4), 513 (2020). 32802799 10.31661/jbpe.v0i0.1912-1027 PMC7416093

[38] Alsmariy R., Healy G., Abdelhafez H.: Predicting cervical cancer using machine learning methods. International Journal of Advanced Computer Science and Applications 11(7) (2020).

[39] Rayavarapu K., Krishna K.K.: Prediction of cervical cancer using voting and dnn classifiers. In: 2018 International Conference on Current Trends Towards Converging Technologies (ICCTCT), pp. 1–5 (2018). IEEE.

[40] Tseng C.-J., Lu C.-J., Chang C.-C., Chen G.-D.: Application of machine learning to predict the recurrence-proneness for cervical cancer. Neural Computing and Applications 24(6), 1311–1316 (2014).

[41] Lu J., Song E., Ghoneim A., Alrashoud M.: Machine learning for assisting cervical cancer diagnosis: An ensemble approach. Future Generation Computer Systems 106, 199–205 (2020).

[42] Akter L., Islam M., Al-Rakhami M.S., Haque M., et al.: Prediction of cervical cancer from behavior risk using machine learning techniques. SN Computer Science 2(3), 1–10 (2021).

[43] Alam T.M., Khan M.M.A., Iqbal M.A., Abdul W., Mushtaq M.: Cervical cancer prediction through different screening methods using data mining. IJACSA) International Journal of Advanced Computer Science and Applications 10(2) (2019).

[44] Wagholikar K.B., MacLaughlin K.L., Casey P.M., Kastner T.M., Henry M.R., Hankey R.A., et al.: Automated recommendation for cervical cancer screening and surveillance. Cancer informatics 13, 14035 (2014). doi: 10.4137/CIN.S14035 25368505 PMC4214690

[45] Nuranna L., Aziz M.F., Cornain S., Purwoto G., Purbadi S., Budiningsih S., et al: Cervical cancer prevention program in jakarta, indonesia: See and treat model in developing country. Journal of gynecologic oncology 23(3), 147–152 (2012). doi: 10.3802/jgo.2012.23.3.147 22808356 PMC3395009

[46] Quercia K., Tran P.L., Jinoro J., Herniainasolo J.L., Viviano M., Vassilakos P., et al: A mobile health data collection system for remote areas to monitor women participating in a cervical cancer screening campaign. Telemedicine and e-Health 24(4), 277–282 (2018). 10.1089/tmj.2017.0146 28846504

[47] Gallay C., Girardet A., Viviano M., Catarino R., Benski A.-C., Tran P.L., et al: Cervical cancer screening in low-resource settings: a smartphone image application as an alternative to colposcopy. International journal of women’s health 9, 455 (2017). doi: 10.2147/IJWH.S136351 28790867 PMC5489054

[48] Labani S., Asthana S., Sodhani P., Gupta S., Bhambhani S., Pooja B., et al: Carehpv cervical cancer screening demonstration in a rural population of north india. European Journal of Obstetrics & Gynecology and Reproductive Biology 176, 75–79 (2014). 24685404 10.1016/j.ejogrb.2014.03.006

[49] Munoz-Zuluaga C.A., Gallo-Pérez J.D., Pérez-Bustos A., Orozco-Urdaneta M., Druffel K., Cordoba-Astudillo L.P., et al.: Mobile applications: Breaking barriers to early breast and cervical cancer detection in underserved communities. JCO Oncology Practice 17(3), 323–335 (2021). doi: 10.1200/OP.20.00665 33417491 PMC8258140

[50] Mingo A.M., Panozzo C.A., DiAngi Y.T., Smith J.S., Steenhoff A.P., Ramogola-Masire D., et al: Cervical cancer awareness and screening in botswana. International Journal of Gynecologic Cancer 22(4) (2012). doi: 10.1097/IGC.0b013e318249470a 22367370 PMC4437542

[51] Coronado Interis E., Anakwenze C.P., Aung M., Jolly P.E.: Increasing cervical cancer awareness and screening in jamaica: Effectiveness of a theory-based educational intervention. International journal of environmental research and public health 13(1), 53 (2016). 10.3390/ijerph13010053 PMC4730444 26703641

[52] Wright T.C. Jr, Kuhn L.: Alternative approaches to cervical cancer screening for developing countries. Best practice & research Clinical obstetrics & gynaecology 26(2), 197–208 (2012). doi: 10.1016/j.bpobgyn.2011.11.004 22385539

[53] Peterson C.W., Rose D., Mink J., Levitz D.: Real-time monitoring and evaluation of a visual-based cervical cancer screening program using a decision support job aid. Diagnostics 6(2), 20 (2016). doi: 10.3390/diagnostics6020020 27196932 PMC4931415

[54] Naz M.S.G., Kariman N., Ebadi A., Ozgoli G., Ghasemi V., Fakari F.R.: Educational interventions for cervical cancer screening behavior of women: a systematic review. Asian Pacific journal of cancer prevention: APJCP 19(4), 875 (2018). 29693331 10.22034/APJCP.2018.19.4.875 PMC6031778

[55] Naz M.S.G., Kariman N., Ebadi A., Ozgoli G., Ghasemi V., Fakari F.R.: Educational interventions for cervical cancer screening behavior of women: a systematic review. Asian Pacific journal of cancer prevention: APJCP 19(4), 875 (2018). 29693331 10.22034/APJCP.2018.19.4.875 PMC6031778

[56] Hoque M.R., Haque E., Karim M.R.: Cervical cancer in low-income countries: A bangladeshi perspective. International Journal of Gynecology & Obstetrics 152(1), 19–25 (2021). doi: 10.1002/ijgo.13400 32989750

[57] Vu M., Yu J., Awolude O.A., Chuang L.: Cervical cancer worldwide. Current problems in cancer 42(5), 457–465 (2018). doi: 10.1016/j.currproblcancer.2018.06.003 30064936

[58] Munoz N., Bosch F., De Sanjosé S., Shah K.: The role of hpv in the etiology of cervical cancer. Mutation Research/Fundamental and Molecular Mechanisms of Mutagenesis 305(2), 293–301 (1994). doi: 10.1016/0027-5107(94)90249-6 8121439

[59] Canfell K.: Towards the global elimination of cervical cancer. Papillomavirus research 8, 100170 (2019). doi: 10.1016/j.pvr.2019.100170 31176807 PMC6722296

[60] Ward Z.J., Grover S., Scott A.M., Woo S., Salama D.H., Jones E.C., et al.: The role and contribution of treatment and imaging modalities in global cervical cancer management: survival estimates from a simulation-based analysis. The Lancet Oncology 21(8), 1089–1098 (2020). doi: 10.1016/S1470-2045(20)30316-8 32758463 PMC7574952

[61] Ponniah H.S., Shah V., Rad A.A., Vardanyan R., Miller G., Malawana J.: Theatres without borders: a systematic review of the use of intraoperative telemedicine in low-and middle-income countries (lmics). BMJ Innovations 7(4) (2021).

[62] Singh S., Fletcher G.G., Yao X., Sussman J.: Virtual care in patients with cancer: a systematic review. Current Oncology 28(5), 3488–3506 (2021). doi: 10.3390/curroncol28050301 34590602 PMC8482228

[63] Rodriguez-Villa E., Naslund J., Keshavan M., Patel V., Torous J.: Making mental health more accessible in light of covid-19: Scalable digital health with digital navigators in low and middle-income countries. Asian journal of psychiatry 54, 102433 (2020). doi: 10.1016/j.ajp.2020.102433 33271713 PMC11694478

[64] Cervical cancer—apps on Google Play. Google (2020). https://play.google.com/store/apps/details?id=com.newandromo.dev338923.app609995.

[65] Cervical cancer—apps on Google Play. Google (2021). https://play.google.com/store/apps/details?id=com.Cervical.cancer.

[66] Cervical cancer guide—apps on google play. Google (2020). https://play.google.com/store/apps/details?id=com.andromo.dev667101.app686769.

[67] Cervical cancer—apps on Google Play. Google (2021). https://play.google.com/store/apps/details?id=ae.index.app.focp.

[68] Cervical cancer—apps on Google Play. Google (2019). https://play.google.com/store/apps/details?id=com.bmooble.android.app.cervicalCancer.

[69] Cervical cancer—news—apps on Google Play. Google (2020). https://play.google.com/store/apps/details?id=com.buzzato.cervicalcancer.

[70] Cervical cancer tracker—apps on Google Play. Google (2020). https://play.google.com/store/apps/details?id=com.tafutaa.hellen.

[71] Cervical cancer risk assessmen—apps on Google Play. Google (2020). https://play.google.com/store/apps/details?id=com.proactiffhealthcare.cervicalcancer.

[72] Mukakalisa I., Bindler R., Allen C., Dotson J.: Cervical cancer in developing countries: effective screening and preventive strategies with an application in Rwanda. Health care for women international 35(7–9), 1065–1080 (2014). doi: 10.1080/07399332.2014.909433 24750113

[73] Okpara C.V., Anselm A.U., Felix T.O., Omowale A., Gever V.C.: The moderating role of colour in modelling the effectiveness of covid-19 youtube animated cartoons on the health behaviour of social media users in nigeria. Health promotion international 36(6), 1599–1609 (2021). doi: 10.1093/heapro/daab001 33729511 PMC7989244

[74] Pervez M.S., Dutta P.: Cancer survivorship in the digital era: Special reference to facebook health groups. Forest Chemicals Review, 1401–1410 (2022).

[75] Tenkorang E.Y., Gyimah S.O., Maticka-Tyndale E., Adjei J.: Superstition, witchcraft and hiv prevention in sub-saharan africa: the case of ghana. Culture, health & sexuality 13(9), 1001–1014 (2011). doi: 10.1080/13691058.2011.592218 21714753

[76] Volkova O., Smirnova E.: Improvement of organizational approaches to regular medical checkup service in the metropolitan healthcare system. Problems of Social Hygiene, Public Health and History of Medicine 28, 1094–1100 (2020). doi: 10.32687/0869-866X-2020-28-s2-1094-1100 33219764

[77] Keir A., Bamat N., Patel R.M., Elkhateeb O., Roland D.: Utilising social media to educate and inform healthcare professionals, policy-makers and the broader community in evidence-based healthcare. BMJ Evidence-Based Medicine 24(3), 87–89 (2019). doi: 10.1136/bmjebm-2018-111016 30049686

[78] Benstead L.J.: Conceptualizing and measuring patriarchy: The importance of feminist theory. Mediterranean Politics 26(2), 234–246 (2021).

[79] Faruk H.: Health inequalities and underdeveloped societies socioeconomics; critical human security with a significant focus on africa and the middle east. InterConf (2021).

[80] Susman P., O’Keefe P., Wisner B.: Global disasters, a radical interpretation. In: Interpretations of Calamity, pp. 263–283. Routledge,??? (2019).

[81] Gokagglers: Cervical cancer risk classification (2017). https://www.kaggle.com/datasets/loveall/cervical-cancer-risk-classification.

[82] Brownlee J.: How to choose a feature selection method for machine learning. Machine Learning Mastery 10 (2019).

[83] Del Cueto M., Troisi A.: Determining usefulness of machine learning in materials discovery using simulated research landscapes. Physical Chemistry Chemical Physics 23(26), 14156–14163 (2021). doi: 10.1039/d1cp01761f 34079968

[84] Suetens P.: Fundamentals of Medical Imaging. Cambridge university press,??? (2017).

[85] Nawir M., Amir A., Yaakob N., Lynn O.B.: Effective and efficient network anomaly detection system using machine learning algorithm. Bulletin of Electrical Engineering and Informatics 8(1), 46–51 (2019)

[86] Yang Q., Liu Y., Cheng Y., Kang Y., Chen T., Yu H.: Federated learning. Synthesis Lectures on Artificial Intelligence and Machine Learning 13(3), 1–207 (2019).

[87] Ganggayah M.D., Taib N.A., Har Y.C., Lio P., Dhillon S.K.: Predicting factors for survival of breast cancer patients using machine learning techniques. BMC medical informatics and decision making 19(1), 1–17 (2019) 30902088 10.1186/s12911-019-0801-4 PMC6431077

[88] Katusiime J., Pinkwart N.: A mobile app for illiterate and semi-illiterate pregnant women-a user centered approach. In: IFIP Conference on Human-Computer Interaction, pp. 617–620 (2019). Springer.

